# Forensisch-psychiatrische Patient:innen mit Unterbringungsgrundlage § 63 StGB in Mecklenburg-Vorpommern – Ergebnisse einer Machbarkeitsstudie zur Entwicklung einer Stichtagserhebung im Maßregelvollzug

**DOI:** 10.1055/a-2567-6808

**Published:** 2025-06-02

**Authors:** Katja Köppen, Peggy Walde, Birgit Völlm

**Affiliations:** 1 Klinik für Forensische Psychiatrie, Universitätsmedizin Rostock, Zentrum für Nervenheilkunde, Rostock; 2 LVR-Institut für Forschung und Bildung, Bereich Forschung, Köln

**Keywords:** Forensische Psychiatrie, Maßregelvollzug, Stichtagserhebung, Machbarkeitsstudie, Forensic psychiatry, Forensic mental health, Annual survey, Pilot study

## Abstract

Um die Umsetzbarkeit einer deutschlandweiten Erhebung der Patientenpopulation im Maßregelvollzug sicherzustellen, wurde im Rahmen einer Machbarkeitsstudie eine Vollerhebung aller Patient:innen mit Unterbringungsgrundlage § 63 StGB in Mecklenburg-Vorpommern durchgeführt. Mitarbeitende teilnehmender Kliniken füllten einen Onlinefragebogen zu biografischen, klinischen und rechtlichen Daten aus, die deskriptiv ausgewertet wurden. Der Dateneingabeprozess wurden begleitet und kritisch überprüft. Es wurden Daten von N=145 Patient:innen erhoben (M=42 Jahre). Die häufigste Diagnose war Schizophrenie, die Hälfe der Patient:innen hatte eine komorbide Substanzkonsumstörung. Die Unterbringungsdauer war länger als im gesamtdeutschen Durchschnitt. Auffallend war der geringe Anteil evidenzbasierter Behandlungsangebote. Die Pilotstudie zeigt, dass die Durchführung einer bundesweiten Stichtagserhebung praktisch machbar ist und die Herausforderungen lösbar sind. Ein inhaltlicher Fokus sollte dabei auf Therapieangeboten und deren Einfluss auf Verlaufsindikatoren liegen.

## Abstract (en)

In order to ensure the feasibility of a nationwide annual survey of patients in forensic psychiatry, a pilot study was conducted to collect data from the entire § 63 StGB patient population of Mecklenburg-Western Pomerania. Staff of the participating hospitals completed an online questionnaire on biographical, clinical and legal data that were analyzed descriptively. The data collection process was monitored and critically reviewed.Data on 145 patients (mean age 42 years) was collected. Schizophrenia was most frequently diagnosed, about half of the patients had a comorbid substance use disorder. Length of stay was longer compared to the German average. Notable is the low proportion of evidence-based treatment programs.The pilot showed that a nationwide survey of forensic-psychiatric patients is feasible and challenges can be overcome. The focus should be on therapies offered and their impact on otcomes.

## Einleitung

In der forensischen Psychiatrie sind Menschen untergebracht, die aufgrund einer psychischen Erkrankung eine Straftat im Zustand der Schuldunfähigkeit oder verminderten Schuldfähigkeit begangen haben und von denen weitere erhebliche rechtswidrige Taten zu erwarten sind (§ 63 StGB). Die Unterbringung ist unbefristet, sehr kostenintensiv 1 und greift in besonderem Maße in die freiheitlichen Grundrechte ein. Therapeutische Konzepte und Behandlungsorganisationen sollten daher zwingend von der aktuellen Evidenzlage geleitet sein. Allerdings fehlt es derzeit selbst an grundlegenden Informationen zu dieser Patientenpopulation in Deutschland 2 3 . Die Beschreibung und wissenschaftliche Erforschung der Population ist jedoch notwendig, um zu evaluieren, ob die Unterbringung überhaupt ihren Zweck, nämlich das Risiko erneute Straftaten zu reduzieren, erfüllt und welche Behandlungskonzepte hierfür am effizientesten sind 4 .

Die jährliche Stichtagserhebung „CONNECT“ (‘ Co llaboration to establish a n atio n al databas e on the c riminological and t reatment outcomes of forensic psychiatric patients in Germany’) soll diese Datenlücke schließen, die Effizienz von Behandlungskonzepten evaluieren und Prädiktoren für Behandlungserfolg identifizieren. Die Erhebung soll belastbare und objektive Daten generieren, die der Forschung, der Politik, der Gesellschaft und den Betroffenen selbst dienen. Die erhebliche Belastung der Betroffenen durch die Einschränkung ihrer Grundrechte, der Anspruch der Öffentlichkeit auf einen effektiven Schutz durch wirksame Behandlung sowie die aktuellen Diskussionen um durch unzureichende personelle und materielle Ausstattung der Maßregelvollzugskliniken in Deutschland erleichterte gewalttätige intramurale Übergriffe 3 machen die Erhebung dringend erforderlich. Für die Sicherstellung der Umsetzbarkeit der geplanten deutschlandweiten Erhebung wurde im Rahmen einer Machbarkeitsstudie eine Vollerhebung aller Patient:innen mit Unterbringungsgrundlage § 63 StGB in den drei forensischen Kliniken Mecklenburg-Vorpommerns durchgeführt.

## Methode

### Durchführung der Erhebung

Mitarbeitende der Kliniken – administrative Kräfte und/oder die fallführenden Therapeut:innen – füllten im Jahr 2023 einen Online-Fragebogen aus. Der Stichtag war der 31.12.2022, d. h. alle Angaben beziehen sich auf das Jahr 2022. Es wurden Patient:innen erfasst, die zum Stichtag entweder a) in der Klinik untergebracht waren, b) im letzten Kalenderjahr mit richterlichem Beschluss entlassen wurden oder c) im letzten Kalenderjahr in eine andere Maßregelvollzugseinrichtung verlegt wurden. Es wurden vor allem Patientendaten genutzt, die im Rahmen der Aufnahmediagnostik bereits erfragt wurden, aber auch Therapeuteninformationen. Die Patient:innen wurden also nicht direkt befragt. Die Daten wurden über die Software *SoSci Survey* an die Forschenden übermittelt 5 . Anfang 2023 wurden zwei Pretest-Runden durchgeführt, um an einer kleinen Stichprobe die technische Umsetzung und Verständlichkeit des Online-Fragebogens zu testen. Zwischen Juni und November 2023 fand die eigentliche Erhebungsphase statt. Erfasst wurden biografische Daten, klinische Anamnese sowie die Deliktanamnese (Basismodul). Zusätzlich erhoben wir Daten zum Behandlungsverlauf, z. B. sicherheitsrelevante Vorfälle und eingesetzte Zwangsmaßnahmen, sowie Behandlungsprogramme, Medikation und Lockerungen (klinisches Forschungsmodul). Im Entlassungsmodul wurde Daten von Patient:innen erfasst, die bis zum Stichtag entlassen worden sind, z. B. Daten zum sozialen Empfangsraum oder gerichtliche Weisungen.

### Ethik und Datenschutz

Die Studie erhielt ein positives Ethikvotum durch die zuständige Ethik-Kommission (EK Universitätsmedizin Rostock/A 2021-0003). Zudem stimmte das zuständige Ministerium der Studie zu und stellte fest, dass das öffentliche Interesse an der Durchführung des Forschungsvorhabens die schutzwürdigen Belange der Patient:innen erheblich überwiegt und somit die Datenverarbeitung auch ohne die Einwilligung der Betroffenen möglich ist 1 . Bedingt durch die Sensibilität der Daten wurden weitgehende Maßnahmen getroffen, um die Vertraulichkeit zu gewährleisten. Alle Informationen werden in pseudonymisierter Form erhoben, so dass niemals Klarnamen oder Geburtsdaten übermittelt werden. Die Zuordnung von Patientennamen und Probandencodes verblieb in den teilnehmenden Einrichtungen. In der Hauptstudie werden die Kliniken zudem gebeten, keine Daten zu übermitteln, die leicht identifizierbare/öffentlich bekannte Patient:innen (High Profile Patient:innen) beschreiben.

### Datenanalyse

Zur Auswertung wurden deskriptive Statistiken sowie Chi-Quadrat-Tests und Maße für die Effektstärke mit *STATA 17* berechnet.

## Ergebnisse

### Stichprobe

Es wurden Daten von *N* =145 Patient:innen erhoben. Davon wurden 9% (n=13) innerhalb des Kalenderjahres 2022 mit richterlichem Beschluss entlassen. Die restlichen Patient:innen waren zum Stichtag im Durchschnitt seit 108 Monaten in der aktuell erfassten Unterbringung ( *SD* ±87,4, *Md* =79, Spannweite=8–388 Monate).

### Basismerkmale

Die Patient:innen waren überwiegend männlich (94%) und durchschnittlich 42 Jahre alt ( *SD* ±12,9, *Md* =39, Spannweite=20–84 Jahre). Einen Migrationshintergrund wiesen 14% auf. 16,5% hatten einen Förderschulabschluss, 37,2% einen Hauptschulabschluss, 19,3% die mittlere Reife und 6,2% ein (Fach-) Abitur. 65% hatten keinen beruflichen Bildungsabschluss und ein ebenso großer Anteil war zum Zeitpunkt des Anlassdeliktes nicht erwerbstätig. Die Diagnosen wurden nach ICD-10 klassifiziert; Mehrfachangaben waren möglich. 47% waren von einer Schizophrenie bzw. schizotypen oder wahnhaften Störung (F20-29) betroffen, 30% wiesen eine Intelligenzminderung auf (F70-79). Bei 19% wurden Störungen der Sexualpräferenz (F65) diagnostiziert, 13% wiesen andere Persönlichkeits- und Verhaltensstörungen auf (F60-69). Die Hälfte der Patient:innen war von einer Substanzkonsumstörung betroffen (F10-F19).

### Klinische Anamnese

56,5% der Patient:innen waren bereits vor dem aktuellen Aufenthalt mindestens einmal in stationärer allgemeinpsychiatrischer; 16% in stationärer suchtmedizinischer Behandlung. Fast ein Viertel (22%) hatte bereits einen Suizidversuch hinter sich. Viele der Patient:innen waren Belastungsfaktoren in Kindheit/Jugend ausgesetzt: Die Hälfte wuchs mit Eltern auf, die von Alkohol oder anderen Substanzen abhängig waren. 41,4% hatten körperlichen, emotionalen oder sexuellen Missbrauch erlebt, oftmals durch Sorgeberechtigte.

### Deliktanamnese und juristische Merkmale

Ein Drittel wurde vor der aktuellen Maßregel bereits mindestens einmal strafrechtlich verurteilt; 17,9% wiesen mindestens eine vorherige abgeschlossene Maßregelvollzugsunterbringung auf. Im Durchschnitt waren pro Patient:in 6 Einträge im Bundeszentralregister aufgezeichnet ( *SD* ±5,6, *Md* =4,5, Spannweite=1–30). Das mittlere Alter bei der ersten Delinquenz lag bei 18,3 Jahren ( *SD* ±4,4, *Md* =17, Spannweite=14–30). Bei etwa der Hälfte der Patient:innen wurde zum Tatzeitpunkt eine Schuldunfähigkeit, bei der anderen Hälfte eine verminderte Schuldfähigkeit festgestellt. Von den 145 untersuchten Personen wurden 221 Anlassdelikte verübt. Es dominieren Körperverletzungs- und sonstige Gewaltdelikte (40% und 23%), Sexualdelikte ggü. Minderjährigen (17%), versuchte Tötungsdelikte (15%) und Brandstiftung (12%).

### Behandlungsbezogene Merkmale

**Abb.1:**
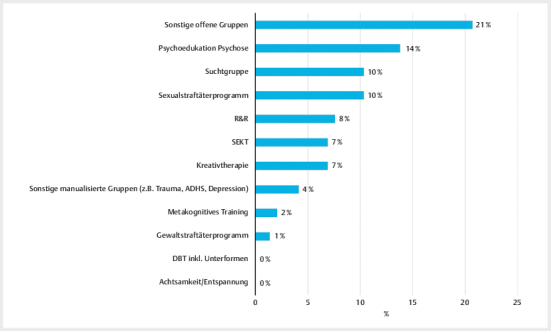
Behandlungsprogramme: Teilnahme an Gruppentherapien (Mehrfachantworten möglich).

In Abb. 1 werden die erfassten Gruppentherapien im Jahr 2022 dargestellt 2 . Mehrfachangaben waren möglich.

Auffallend ist, dass nur ein geringer Anteil an Patient:innen evidenzbasierte Gruppentherapien angeboten bekam. Am häufigsten aus der Gruppe der manualisierten Therapien nahmen Patient:innen im Verlauf ihrer Unterbringung das Angebot „Psychoedukation Psychose“ in Anspruch (14%). Es wurden vor allem „sonstige offene Gruppenangebote“ erwähnt (21%), hier insbesondere sogenannte „Stationsgruppen“. Einzeltherapien waren zumeist verhaltenstherapeutisch ausgerichtet (74%). 13% nutzten kreativtherapeutische Angebote und 17% nicht weiter differenzierte „sonstige“ Angebote, darunter wurden vor allem die systemische Einzeltherapie und die Psychose-Sprechstunde genannt. Um den Therapieerfolg zu evaluieren, nahmen die zuständigen Behandelnden eine fachliche Einschätzung auf einer Likert-Skala von 1 (Stimme gar nicht zu) bis 5 (Stimme voll zu) in Bezug auf Therapieteilnahme, Opferempathie und Tatverantwortung vor. Die Behandelnden bewerteten die Regelmäßigkeit der Teilnahme ihrer Patient:innen an der Therapie überwiegend gut ( *M* =3,6, *SD* ±1,3), sahen jedoch das Verständnis der Patient:innen für die Tat und ihre Opfer ( *M* =2,6, *SD* ±1,2) sowie die Bereitschaft, Verantwortung zu übernehmen ( *M* =2,4, *SD* ±1,3), kritisch.

**Abb. 2:**
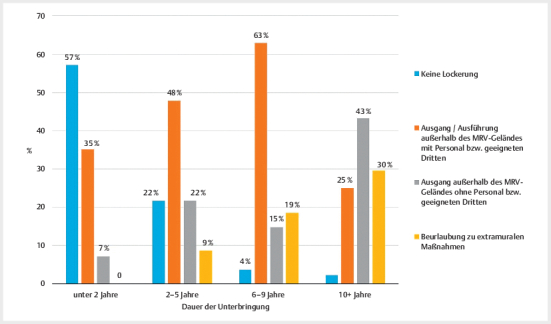
Vollzugslockerung nach Dauer der aktuellen Maßregel (Beurlaubung zu extramuralen Maßnahmen enthält Belastungsurlaub und Probewohnen/Langzeitbeurlaubung).

Abb. 2 zeigt die Lockerungen nach Dauer der aktuellen Maßregel 3 . Die Angaben beziehen sich auf die Bestandspatient:innen, deren Maßregel zum Stichtag noch nicht beendet war. Mit einer höheren Aufenthaltsdauer gingen auch höhere Lockerungsstufen einher (n=131; Χ²=47,93; p<.001; Spearman’s *ρ* =0,5). Während ca. ein Drittel der Patient:innen, die weniger als 2 Jahre untergebracht waren, begleiteten Ausgang hatten, waren dies schon fast zwei Drittel bei einer Aufenthaltsdauer von 6–9 Jahren. Unbegleitete Lockerungen betrafen insbesondere Patient:innen mit längerer Verweildauer (>10 Jahre). Diese Patientengruppe lebt zudem zu einem hohen Anteil bereits im Probewohnen/der Langzeitbeurlaubung. Interessant ist die relativ hohe Zahl an Personen, die auch nach längerer Unterbringungsdauer noch keinen Ausgang erhalten hatten (22% bei einer Unterbringungsdauer von 2–5 Jahren).

## Diskussion

### Inhaltliche Ergebnisse

Die Auswertung einer Vollerhebung von 145 Personen im Maßregelvollzug Mecklenburg-Vorpommerns zeigt, dass es sich bei der Patientenpopulation um eine besonders vulnerable Gruppe handelt. Die Anzahl derjenigen mit Migrationshintergrund lag etwas über dem Durchschnitt Mecklenburg-Vorpommerns (14% vs. 10,4%), der Anteil der Patient:innen ohne Schulabschluss war dreimal so hoch wie in der Gesamtbevölkerung des Bundeslandes (31% vs. 10,6%) 6 . Viele hatten keine berufliche Ausbildung, waren ohne Einbindung in den Arbeitsmarkt und wiesen eine längere Vorgeschichte an psychiatrischen Vorbehandlungen, Substanzmissbrauch und Hafterfahrungen auf. Die häufigsten psychiatrischen Diagnosen waren Schizophrenie (47%) und Persönlichkeits- und Verhaltensstörungen (32%). Dieser Befund korrespondiert mit älteren Daten aus einer deutschlandweiten Erhebung von § 63 StGB-Patient:innen 7 . Höher lag in unserer Studie der Anteil der Patient:innen mit Intelligenzminderungen (30% vs. 10%). Aktuelle Daten aus Baden-Württemberg verzeichnen zudem einen höheren Anteil an Störungen aus dem schizophreniformen Spektrum (über 70%; 8 ). Die Gründe hierfür können nur vermutet werden. Ein Grund kann sein, dass für unsere Studie die Diagnosen zum Erhebungszeitpunkt erfasst wurden, während in den anderen Studien die psychiatrische Hauptdiagnose im Urteil zugrunde lag. Je nach Definition (nur Primär- oder auch komorbide Diagnosen), Erhebungsjahr und Bundesland schwanken z. B. die Anteile der Untergebrachten mit einer Intelligenzminderung unter allen im deutschen Maßregelvollzug Untergebrachten zwischen 8% und 22% 9 .

Durch ein breites Spektrum an Therapieformen sollen die Patient:innen auf ein sucht- und straffreies Leben vorbereitet werden und wieder in die Gesellschaft integriert werden. Die Erhebung der angebotenen Therapien gestaltete sich aufgrund der zahlreichen möglichen Therapieformen bei fehlender Standardisierung der forensisch-psychiatrischen Behandlung schwierig. Bei der Zuordnung der angebotenen Therapien zu den von uns gebildeten Kategorien fiel der geringe Anteil an Patient:innen auf, die an evidenzbasierten Gruppentherapien teilgenommen hatten, wie z. B. dem kognitiv ausgerichteten Behandlungsprogramm „Reasoning and Rehabilitation“ (R&R; 10 ). Die Behandlungsinhalte nicht-standardisierter Therapien sind unklar und schwierig zu beurteilen.

Auffallend war eine längere Unterbringungsdauer in Mecklenburg-Vorpommern im Vergleich zum gesamtdeutschen Durchschnitt (ohne Bayern und Baden-Württemberg, vgl. 11 ). Dies passt zu den von den Therapeut:innen angegebenen Zweifeln im Hinblick auf relevante Therapiefortschritte trotz zufriedenstellender Teilnahme. Die geringe Anzahl von Patient:innen, die auch nach langer Unterbringungsdauer die Klinik unbegleitet verlassen darf, deutet darauf hin, dass hier vonseiten der Teams ein anhaltendes Risiko gesehen wird. Diese Ergebnisse sollen Anlass sein, die eingesetzten Therapiekonzepte kritisch zu prüfen, mit dem Ziel, eine höhere Effizienz zu erreichen.

### Implikationen für die Haupterhebung

Die vorliegende Machbarkeitsstudie befasste sich vor allem mit der Frage, ob eine größere Datenerhebung im Bereich des Maßregelvollzuges realisierbar ist. Sie trug maßgeblich dazu bei, die Abläufe und Prozesse der Dateneingabe kritisch zu prüfen und notwendige Änderungen und Verbesserungen zu implementieren. Das Feedback der Mitarbeitenden der Kliniken und eigene Beobachtungen deuteten auf die folgenden wichtigen Faktoren für den reibungslosen Verlauf einer größer angelegten Stichtagserhebung hin:

1. Gute Organisation und feste Absprachen innerhalb der Kliniken
  Insbesondere muss im Vorfeld geklärt werden, wer für welchen Teil der Dateneingabe und welche Patient:innen zuständig ist. Ansonsten besteht die Gefahr von fehlenden oder doppelten Eintragungen, wenn Patient:innen im untersuchten Kalenderjahr auf mehreren Stationen oder von mehreren Therapeut:innen betreut wurden.
2. Möglichst digitale Organisation der Patientenakten
  Für die Erhebung werden vor allem routinemäßig erhobene Daten verwendet. Am hilfreichsten sind elektronisch vorliegende Patientenakten, was jedoch auch heutzutage noch nicht überall der Fall ist. Zusätzlich kommt es durch die unterschiedliche Operationalisierung von Daten zu Herausforderungen bei der Überführung der Daten. Eine direkte Kommunikation mit den Datenverantwortlichen der einzelnen Kliniken hilft, die Variablenoperationalisierung abzustimmen und im Idealfall die klinikinterne Basisdokumentation auf die Anforderungen der Studie einzurichten und dementsprechend anzupassen.
3. Zeitliche und personelle Ressourcenmaximierung
  Viele Kliniken verfügen nicht durchgehend über genügend Kapazitäten, um die Erhebung durchzuführen. Es wird daher den Kliniken ermöglicht, nur an einzelnen Studienmodulen teilzunehmen.
4. Offene Kommunikation mit den Entscheidungsträgern

Um die Erhebung durchführen zu können, muss auf Grund der unterschiedlichen länderrechtlichen Regelungen die Erlaubnis zur Durchführung von verschiedenen Akteuren eingeholt werden: den einzelnen Kliniken, den verantwortlichen Ministerien bzw. Fachaufsichten, den jeweiligen Datenschutzbeauftragten sowie den Klinikträgern. Dafür muss genügend Zeit eingeplant werden. In der Nachbetrachtung ist die Einholung der ministeriellen Genehmigung der wichtigste Schritt. Ohne Zustimmung der zuständigen Fachaufsichten sehen sich die meisten Kliniken, selbst bei Teilnahmebereitschaft, nicht in der Lage, einer Erhebung ihrer Patientendaten zuzustimmen.

## Konsequenzen für Klinik und Praxis

- Eine Erhebung von Basisdaten der Patient:innen mit Unterbringungsgrundlage § 63 StGB ist praktisch möglich, die bestehenden Herausforderungen sind lösbar.
- Eine gute und übersichtlich geführte Dokumentation erhöht die Datenqualität und somit die inhaltliche Aussagekraft. Eine klinikinterne digitale Basisdokumentation erleichtert die Datenübertragung und kann nach Absprache für eine (pseudonymisierte) Verknüpfung mit dem Erhebungsinstrument genutzt werden.
- Durch die Erhebung der unterschiedlichen Behandlungsangebote und -elemente kann eine einheitliche Zuordnung der Therapieangebote über Kliniken hinweg gewährleistet werden, um so eine bessere Vergleichbarkeit zu ermöglichen.
- Für das Gelingen der Implementierung der Erhebung ist insbesondere eine gute Planung und Vorbereitung sowie das Committment wichtiger Entscheidungsträger von Bedeutung.

## Fördermittel

Damp Stiftung — http://dx.doi.org/10.13039/501100013859 ; 2021-16

## Footnotes

^1^ Grundlage dafür sind die folgenden Paragraphen: Art. 89 (1) Datenschutz-Grundverordnung – DS-GVO i.V.m. § 27 Bundesdatenschutzgesetz i.V.m. § 9 Landesdatenschutzgesetz – DSG M-V i.V.m. § 27 Landeskrankenhausgesetz – LKHG M-V

^2^ Therapieteilnahme bedeutet entweder a) aktuelle Teilnahme zum Stichtag, b) regulärer Therapieabschluss im Erhebungszeitraum, c) regulärer Therapieabschluss vor dem letzten Kalenderjahr oder d) Teilnahme, aber irreguläres Therapieende im Erhebungszeitraum.

^3^ „Keine Lockerung“ enthält Hofgang innerhalb des umzäunten MRV-Geländes. Ausführung oder Ausgang umfasst alle Ausführungen oder Ausgänge außerhalb des umzäunten MRV-Geländes.
